# Assessment of Frailty by the French Version of the Vulnerable Elders Survey-13 on Digital Tablet: Validation Study

**DOI:** 10.2196/42017

**Published:** 2023-08-02

**Authors:** Victoria Zolnowski-Kolp, Nathavy Um Din, Charlotte Havreng-Théry, Sylvie Pariel, Jacques-Henri Veyron, Carmelo Lafuente-Lafuente, Joel Belmin

**Affiliations:** 1 LivingLab Pratiques en santé dans le grand âge Hôpital Charles Foix Ivry-sur-Seine France; 2 Sorbonne Université and Institut National de la Santé et de la Recherche Médicale (INSERM) Laboratoire d'Informatique Médicale et d'Ingénierie des Connaissances pour la e-Santé (LIMICS) Paris France; 3 Presage Care Paris France; 4 Service de gériatrie ambulatoire Hôpital Charles Foix Ivry-sur-Seine France; 5 Service de gériatrie à orientation cardiovasculaire et neuropsychogériatrique Hôpital Charles Foix Ivry-sur-Seine France; 6 Clinical Epidemiology and Ageing (CEpiA) Unit Institut National de la Santé et de la Recherche Médicale (INSERM) Créteil France

**Keywords:** frailty, Vulnerable Elders Survey-13 (VES-13), elderly, older people, family caregivers, French version, electronic assessment, digital technology, digital health, eHealth, tablet, validity

## Abstract

**Background:**

Frailty assessment is a major issue in geriatric medicine. The Vulnerable Elders Survey-13 (VES-13) is a simple and practical tool that identifies frailty through a 13-item questionnaire completed by older adults or their family caregivers by self-administration (pencil and paper) or by telephone interview. The VES-13 provides a 10-point score that is also a recognized mortality predictor.

**Objective:**

This study aims to design an electronic version of the Echelle de Vulnérabilité des Ainés-13, the French version of the VES-13 (eEVA-13) for use on a digital tablet and validate it.

**Methods:**

The scale was implemented as a web App in 3 different screens and used on an Android tablet (14.0× 25.6 cm). Participants were patients attending the outpatient clinic of a French geriatric hospital or hospitalized in a rehabilitation ward and family caregivers of geriatric patients. They completed the scale twice, once by a reference method (self-administered questionnaire or telephone interview) and once by eEVA-13 using the digital tablet. Agreement for diagnosis of frailty was assessed with the κ coefficient, and scores were compared by Bland and Altman plots and interclass correlation coefficients. User experience was assessed by a self-administered questionnaire.

**Results:**

In total, 86 participants, including 40 patients and 46 family caregivers, participated in the study. All family caregivers had previously used digital devices, while 13 (32.5%) and 10 (25%) patients had no or infrequent use of them previously. We observed no failure to complete the eEVA-13, and 70% of patients (28/40) and no family caregivers needed support to complete the eEVA-13. The agreement between the eEVA-13 and the reference method for the diagnosis of frailty was excellent (κ=0.92) with agreement in 83 cases and disagreement in 3 cases. The mean difference between the scores provided by the 2 scales was 0.081 (95% CI–1.263 to 1.426). Bland and Altman plots showed a high level of agreement between the eEVA-13 and the reference methods and interclass correlation coefficient value was 0.997 (95% CI 0.994-0.998) for the paper and tablet group and 0.977 (95% CI 0.957-0.988) for the phone and tablet groups. The tablet assessment was found to be easy to use by 77.5% (31/40) of patients and by 96% (44/46) of caregivers. Finally, 85% (39/46) of family caregivers and 50% (20/40) of patients preferred the eEVA-13 to the original version.

**Conclusions:**

The eEVA-13 is an appropriate digital tool for diagnosing frailty and can be used by older adults and their family caregivers. The scores obtained with eEVA-13 are highly correlated with those obtained with the original version. The use of health questionnaires on digital tablets is feasible in frail and very old patients, although some patients may need help to use them.

## Introduction

The concept of frailty has been widely studied in aging research for the past decades [1,2]. Frailty is characterized by a vulnerability related to advancing age, along with a reduction in the functional reserves of physiological systems that impairs responses to stress. Older individuals identified as frail are considered vulnerable in terms of their medical and social status, and compared to age-matched nonfrail people, they are more likely to experience geriatric deleterious events in the future [3]. Several studies found that frailty status is a significant predictor of decline in functional independence, onset of geriatric syndromes (such as falls, malnutrition, and depression), but also institutionalization, and even death [4-6]. Thus, the identification of frailty in older adults opens the way to targeted interventions to prevent or delay these geriatric events, whose individual and societal consequences are considerable [7-11]. These issues have been widely emphasized by the World Health Organization, which has initiated proactive programs to promote healthy and active aging [12,13].

The search for frailty is no longer restricted to the field of research, but is becoming part of routine care for an increasing number of geriatric teams, and the question of diagnostic strategies for this condition is clearly raised in these settings and also in primary care. Two instruments, the Fried Frailty Index [2] and the Rockwood Clinical Frailty Scale [14], are reference methods to diagnose frailty in older adults. Many other instruments have been developed to identify frailty in older individuals, and the benefits and limitations of these instruments are under debate [15,16]. However, these are relatively complex tools that are time-consuming and require a high level of geriatric expertise [9], making their use difficult for some geriatric teams and inappropriate for primary care. The Vulnerable Elders Survey-13 (VES-13) is a validated tool to identify frailty in older adults and is a predictor of the risk of functional decline, institutionalization, or mortality [17,18]. This tool is easy-to-use, brief (less than 5 minutes), and can be completed during a face-to-face interview or by telephone with the older individual or with his or her proxy. The scale has 13 simple items or questions, provides a score ranging from 0 to 10, and frailty is diagnosed by a score of 3 or more. The VES-13 can be completed without the need to involve health care staff, and for example, a trained secretary can interview the respondent or supervise the completion of the self-administrated questionnaire. Recently, the original English-language VES-13 has been validated in French, named Echelle de vulnérabilité des aînés-13 (EVA-13), and can now be used to assess frailty in France [19].

With the increasing use of informatics in health care, a number of studies have focused on the design and validation of digital versions of known screening or assessment instruments originally developed on paper. Various electronic media have been used in this research, such as computers, tactile tablets, and smartphones. Digital assessment tools have been developed for pain assessment [20,21], the function of the shoulder joint [22], food intake [23], quality of life measurement [24], dermatological monitoring [25], or erectile function [26]. These studies have documented the reliability of these digital instruments, their equivalence to the original version, and have evaluated the users’ experience with the original version. These digital instruments appear to be useful for screening or assessment because of their accuracy of detection, their ecological aspects, and their speed of execution, which saves time for physicians and can have positive effects on health [27]. Few studies have so far evaluated the feasibility and acceptability of a digital assessment tool in an older population.

This study aims to develop an electronic version of the French version of the VES-13 on a digital tablet. To do this, we will compare the results obtained by the original paper version of the French version of the VES-13 and its digital version, named Echelle électronique de vulnérabilité des ainés-13 (eEVA-13), with a population of older people and their caregivers. We wish to measure the reliability of the digital version in detecting frailty, but also its evaluation and acceptability, compared to a scale already used in its paper version in routine care.

## Methods

### Elaboration of eEVA-13

In this study, eEVA-13 was displayed on an Android tablet with a width of 14.9 cm (5.5 inches), a height of 25.8 cm (10.2 inches), a depth of 1.06 cm (0.42 inches), and a total weight of 515 g. This tablet has a 25.6 cm (10.1 inch) multitouch screen. A secured web application was elaborated to host the data on a personal health data host certified by the Agence Numérique en Santé (Microsoft Azure). It comprised secure web access (strong authentication) through a supervision console [28]. Once the authentication has been successfully completed by the research assistant, he or she identifies the data collector and the latter fills in the questionnaire in accordance with the illustrative input screens provided on the digital tablet. In designing the screens, emphasis was placed on the appearance and clarity of the messages. The answer possibilities are specified for each question, with attention paid to the visibility of the proposed answers.

**Figure 1:**
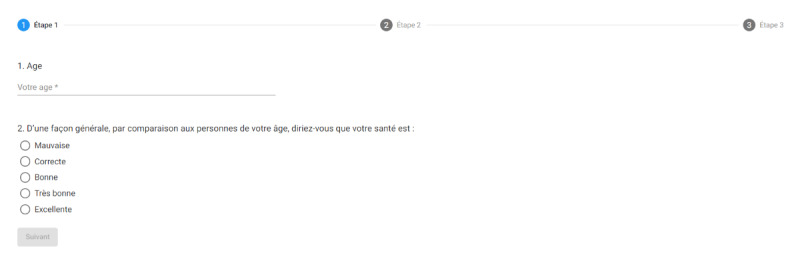
Screen print of the first 2 questions of the Echelle électronique de vulnérabilité des ainés-13 (eEVA-13).

**Figure 2:**
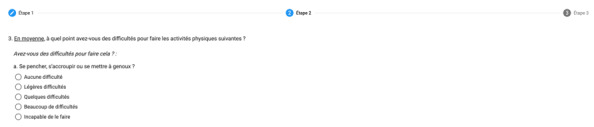
Screen print of the beginning of the next 6 questions of the Echelle électronique de vulnérabilité des ainés-13 (eEVA-13).

The original scale was organized in 3 screens in eEVA-13. The 2 first questions (age group and health status) were displayed on the first screen (Figure 1), the 6 following questions were displayed on the second screen which requires scrolling (Figure 2), and the 5 last items on the third screen, which also require scrolling. For the items on the third screen inquiring about limitations in some physical activities, an additional question (“is it because of your health?”) unfolded for items answered with a limitation. The score was calculated automatically and displayed in a banner at the bottom of the third screen. The cutoff point defining frailty was a score of 3 or higher, as with the paper-and-pencil or telephone interview scales. The research technician prepared the access page for the electronic questionnaire in advance so that when the digital tablet was handed over to the participants, the form was already ready for input. In this access page, the research technician recorded if the respondent was the person assessed or a proxy, and noted an id code for the person assessed and his or her age (years).

### Participants and Randomization

Participants were geriatric patients and family caregivers of geriatric patients of the Hôpital Charles Foix, an academic geriatric hospital located near Paris, France. From March 17, 2021, to June 28, 2021, we recruited consecutive patients attending the outpatient geriatric clinic and inpatients admitted to our rehabilitation ward. Eligible patients were included if they did not experience cognitive impairment defined by a Mini Mental Status Examination score above 22 and if they gave their written consent. During the same period, we also recruited a comparable number of family caregivers of geriatric patients attending the outpatient clinic or being hospitalized.

Each participant was assigned to 1 of 2 groups based on a randomization process stratified on the participant’s status (patient or caregiver). For the reference scale, participants in group 1 completed the self-administered paper version, and those in group 2 completed the scale as a telephone hetero-questionnaire (interview with a research technician). The eEVA-13 and the reference scale were completed within 2 days.

### Data Collection

For all participants, we recorded their age, sex, status (patient or family caregiver), previous use of digital devices, and whether they owned a digital device and if so, what type. Participants were considered occasional users if they used a digital device less than once a week, and frequent users if it was more often. All participants completed 2 scales, the eEVA-13 on a digital tablet and the reference scale (self-administered paper questionnaire for group 1 participants, telephone interview for group 2 participants) in random order. Depending on the degree of comfort with the digital device, we also noted if some people required specific support to fill the eEVA-13. User experience was assessed using a self-administered questionnaire that included eight 5-level Likert-type questions, 2 questions about their preference, and 2 multiple choices questions recording positive and negative aspects of the eEVA-13.

### Ethics Approval

The research has been approved by the French national ethics committee (Comité de Protection des Personnes Ile de France II; 2020-A02331-38). Written informed consent was obtained from all participants. Data were deidentified and processed in accordance with the European Union General data protection regulation. No compensation was provided to participants.

### Statistical Analysis

Characteristic of patients and caregivers were compared using the Student *t* test and chi-square test. Agreement for diagnosis of frailty was assessed by the κ coefficient, and scores obtained at the eEVA-13 were compared to that obtained by the reference tools by Bland and Altman plots and interclass correlation coefficients. Statistics were realized using STATA (version 16.1; StataCorp).

## Results

### Selection and Characteristics of the Participants

**Figure 3:**
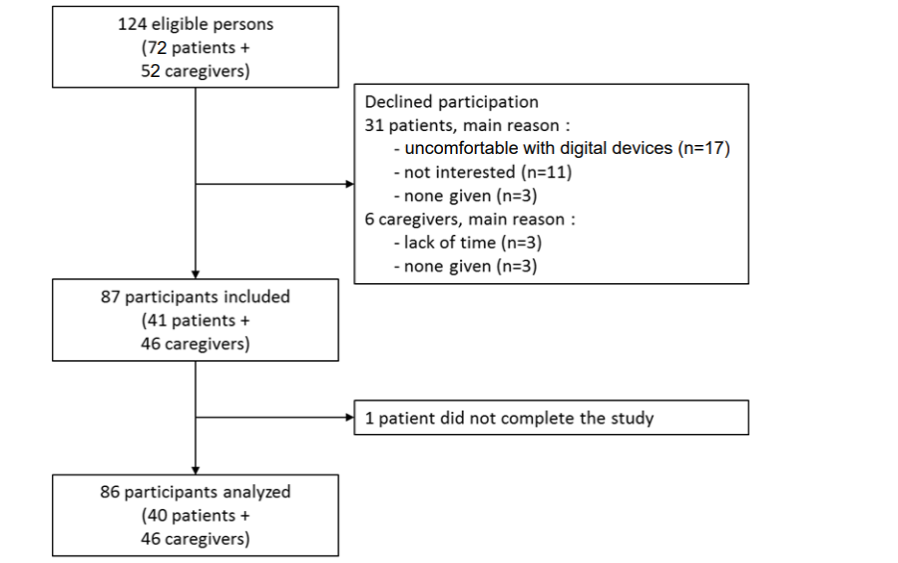
Flowchart for participants’ selection in the study.

During the study period, 124 people were offered the study and 37 of them declined to participate, including 31 patients and 6 family caregivers. Finally, 87 people were included in the study. One of them completed the eEVA-13, but did not complete the reference scale and, therefore, was not included in the analysis. Finally, 86 people completed the study and were included in the analysis. They comprised 40 patients and 46 caregivers (Figure 3).

Participants’ characteristics are shown in Table 1. Family caregivers were significantly younger than the patients (mean 52.8, SD 11.1 years vs mean 79.9, SD 7.5 years, *P*<.001) and were more frequently owners of digital devices and more frequently users of digital devices. Indeed, all the family caregivers say they use digital devices very regularly. By contrast, among the 40 patients, 13 (32.5%) never used digital devices, 10 (25%) were occasional users and 17 (42.5%) were frequent users. All the caregivers had a smartphone, and 39 (87.9%) had a computer and 27 (67.5%) had a digital tablet. Among the patients, 29 had a smartphone (72.5%), 15 (37.5%) a computer, and 4 had a digital tablet (10%).

**Table 1:** Participants’ characteristics.

		Patients (n=40)	Family caregivers (n=46)	*P* values
Age (years), mean (SD; range)		79.9 (7.5; 65-93)	52.8 (11.1; 22-74)	<.001
Female, n (%)		24 (60.0)	31 (67.4)	.501
**Owns digital devices, n (%)**				
	Computer	15 (37.5)	39 (84.7)	.005
	Digital tablet	4 (10.0)	27 (67.5)	<.001
	Smartphone	29 (72.5)	46 (100)	<.001

### Feasibility and Support to Complete the eEVA-13

We observed no failure to complete the eEVA-13. Of the 40 patients, 28 (70%) needed support from the research assistant to complete the eEVA-13, while no caregivers needed support to complete the scale. The support consisted of an explanation of how works the digital tablet (21 cases) and help with navigation from one screen to another (18 cases), scrolling through the screen (23 cases), and selecting answers (24 cases).

### Comparison of eEVA-13 With the Reference Scale

Agreement for the diagnosis of frailty was observed in 83 (96.5%) participants and disagreement was observed in 3 (3.5%) participants. The κ coefficient was 0.92, indicating an excellent level of agreement between eEVA-13 and the reference scales (Table 2).

**Table 2:** Agreement for the diagnosis of frailty provided by the EVA-13^a^ scale (reference method) and the eEVA-13^b^ used on digital tablet (κ=0.92, indicating excellent agreement).

		EVA-13 (reference method)		
		Nonfrail	Frail	Total
**eEVA-13 used on digital tablet**				
	Nonfrail	27	2	29
	Frail	1	56	57
	Total	28	58	86

^a^EVA-13: Echelle de vulnérabilité des aînés-13.

^b^eEVA-13: Echelle électronique de vulnérabilité des ainés-13.

**Figure 4:**
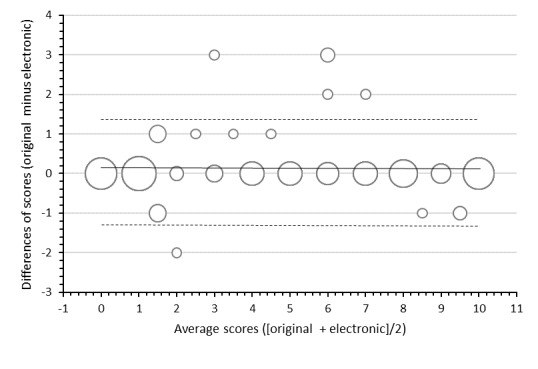
Bland-Altman plot of scores obtained with the Echelle électronique de vulnérabilité des ainés-13 (eEVA-13) and those obtained with the original EVA-13. The solid line represents the bias and the dotted lines the limits of 95% CI.

The equivalence of the scores obtained by eEVA-13 and those obtained by original EVA-13 was assessed by 2 means. First, the intraclass correlation coefficient between eEVA-13 and paper EVA-13 was 0.997 (95% CI 0.994-0.998) and that between eEVA-13 and telephone interview EVA-13 was 0.977 (95% CI 0.957-0.988; Table 3). These results indicate a high degree of agreement between the different versions. Second, we plotted Bland and Altman graphs that showed that the differences between the scores from eEVA-13 and the original scale were small and were not related to the relative value of the scores (Figure 4). The mean difference between the scores provided by the 2 scales was 0.081 (95% CI –1.263 to 1.426).

**Table 3:** Assessment of the equivalence of the scores provided by the eEVA^a^ scale and the original EVA^b^ scale (obtained either by paper questionnaire or by telephone questionnaire).

Comparison	Interclass correlation coefficient (95% CI)	Pearson correlation coefficient	*P* values (Pearson)
Paper versus electronic	0.997 (0.994-0.998)	0.987	<.0001
Telephone versus electronic	0.977 (0.957-0.988)	0.912	<.0001

^a^eEVA-13: Echelle électronique de vulnérabilité des ainés-13.

^b^EVA: Echelle de vulnérabilité des aînés.

### User’s Experience With eEVA-13

User’s experience with eEVA-13 assessed by questionnaire is reported in Table 4. The patients who participated in the study agreed overall (85%) that the original version of the instrument and the electronic version was similar, while among family caregivers this rate was 69%. If they were to complete the scale a second time, 85% of the family caregivers would like to do so in electronic form. As for the patients, 50% of them were divided between the electronic and the original mode. The digital version of the scale was considered easy to use overall (96% of caregivers and 77.5% of patients). The electronic version was considered the most pleasant to complete by 74% of the caregivers, while 67.5% of the patients preferred the original version.

**Table 4:** Users’ experience with the Echelle électronique de vulnérabilité des ainés-13 (eEVA-13). For simplicity, we grouped the responses to the 5 level-Likert scale into 3 levels as described.

Questions			Patients (n=40), n (%)		Caregivers (n=46), n (%)
**How did you find the ease of use of the digital tablet?**					
	Easy or very easy	31 (77.5)		44 (96.0)	
	Nor easy or difficult	6 (15.0)		1 (2.0)	
	Difficult or very difficult	3 (7.5)		1 (2.0)	
**How did you find the ergonomics of the scale on digital tablet?**					
	Good or very good	28 (70.0)		42 (91.0)	
	Average	8 (20.0)		3 (7.0)	
	Poor or very poor	4 (10.0)		1 (2.0)	
**The size of the tablet was appropriate?**					
	Agree or fully agree	33 (82.5)		44 (96.0)	
	Nor agree or disagree	5 (12.5)		0	
	Disagree of fully disagree	2 (5.0)		2 (4.0)	
**The text, the words on the tablet screen were readable enough in your opinion?**					
	Agree or fully agree	35 (87.5)		43 (94.0)	
	Nor agree or disagree	4 (10.0)		2 (4.0)	
	Disagree of fully disagree	1 (2.5)		1 (2.0)	
**The instructions given at each stage of the tablet questionnaire were satisfactory in your opinion?**					
	Agree or fully agree	35 (87.5)		40 (87.0)	
	Nor agree or disagree	5 (12.5)		4 (9.0)	
	Disagree of fully disagree	0		2 (4.0)	
**The questions were clear enough in the tablet version**					
	Agree or fully agree	38 (95.0)		45 (98.0)	
	Nor agree or disagree	1 (2.5)		0	
	Disagree of fully disagree	1 (2.5)		1 (2.0)	
**Did you feel like you were filling the same scale twice?**					
	Agree or fully agree	34 (85.0)		32 (69.0)	
	Nor agree or disagree	2 (5.0)		5 (11.0)	
	Disagree of fully disagree	4 (10.0)		9 (20.0)	
**Do you find it useful to replace the paper scales with a digital version?**					
	Agree or fully agree	22 (55.0)		38 (83.0)	
	Nor agree or disagree	13 (32.5)		5 (11.0)	
	Disagree of fully disagree	5 (12.5)		3 (6.0)	
**Which version of the scale did you find more comfortable?**					
	Paper questionnaire or telephone interview	27 (67.5)		12 (26.0)	
	Digital tablet	13 (32.5)		34 (74.0)	
**If you were asked to complete this scale again in the future, which version would you prefer?**					
	Paper questionnaire or telephone interview	20 (50.0)		7 (15.0)	
	Digital tablet	20 (50.0)		39 (85.0)	
**Can you check off the following terms that apply to this tablet questionnaire?**					
	Practical	22 (55.0)		28 (61.0)	
	Quick	7 (17.5)		12 (26.0)	
	Ergonomic	11 (27.5)		15 (32.5)	
	Secure and ease data processing	11 (27.5)		10 (22.0)	
	Avoid waste	7 (17.5)		6 (13.0)	
	Impersonal tool	4 (10.0)		5 (11.0)	
	Not ergonomic	11 (27.5)		11 (24.0)	
	Not suitable for the older persons	15 (37.5)		10 (22.0)	
	Generates apprehension	9 (22.5)		0	

## Discussion

### Principal Findings

This study shows that the digital version of the eEVA-13 is appropriate for identifying frailty among older adults and that the score it provides is similar to that obtained by the original paper or telephone version of the scale. The feasibility of the eEVA-13 scale was found to be excellent, although some older people who were unfamiliar with the use of digital objects required human assistance to use the digital tablet. According to the participants’ experience questionnaires, the digital scale was found easy to use and was generally appreciated.

Our study was conducted on a special population of frail and very old patients and their relatives. One out of 2 patients was over 80 years old and 2 out of 3 had criteria for frailty. The use of digital tablets for medical assessment has not been studied before in such a population to our knowledge and we observed that it is feasible and well appreciated by the participants.

We observed some differences in usability between patients and family caregivers. Although the digital version was considered easy to use by all participants, the family caregivers stood out by preferring the digital version as being the most pleasant, as well as choosing it for possible future use. The patients were a little more reluctant to use the digital tool to answer the health questionnaire. Among the negative points mentioned by the participants, we can observe that the interest in using the electronic mode by an older population is perceived negatively by 37.5% of the patients and 22% of the caregivers. Indeed, very old patients are sometimes uncomfortable or untrained in the use of a digital tool and prefer to be able to give information in a more traditional way, using a pen and paper or interacting directly with a person. Ten family caregivers spontaneously explained that using the eEVA-13 would be too complex for an older person. These observations are consistent with the inclusion difficulties we encountered during the study. In fact, we collected significantly more refusals from eligible patients (31) than from eligible caregivers (6). Looking at the reasons for refusal among patients (Figure 3), the one that stood out was apprehension about the digital domain, fear of not knowing how to do it, and lack of knowledge in this field. Age and previous familiarity with digital devices seem to be key elements to be considered carefully when proposing the use of an assessment by an electronic instrument.

### Comparison to Prior Work

We found equivalence between the digital version of the scale and the original version of the scale, and our findings are similar to those of other teams that have compared digital and paper versions of other medical tools in other settings [20-26]. Casamali et al [24] studied the use of the World Health Organization quality of life questionnaire on computer in a sample of older adults younger than those in our study (mean age 66 years) and found it to be feasible and to provide similar scores to the original paper instrument. The disadvantages of electronic patient-reported outcomes measures (PROms) have been described in the literature as potentially leading to risks of a “digital divide.” Indeed, it has been observed in some studies that the advanced age of participants was a barrier to completing the electronic version of a questionnaire, compared to the paper version. In the case of older people with cancer or those who are not used to using digital devices, they needed time to get used to the devices or were more likely to encounter barriers when using them if they were not familiar with them beforehand [29].

### Strengths and Limitations

This study was conducted using the recommendations of the International Society for Pharmacoeconomics and Outcomes Research, which emphasize the importance of validating electronic PROms against traditional PROms [30]. In addition, these recommendations provide methodological guidance for documenting equivalence between digital and traditional instruments [30]. As the transition from the original scale to eEVA-13 can be considered as a minor level of modification, our validation study was limited to exploring equivalence and usability, in agreement with the International Society for Pharmacoeconomics and Outcomes Research methodological guidance. We conducted a single-center study with a small sample size of 86 participants. These 2 characteristics represent the main limitations of this work.

### Future Directions

The results obtained in this study show that it is possible to offer the eEVA-13 instrument as a self-questionnaire to family caregivers and older patients. We have started to use it in the waiting room of an outpatient department of a geriatric center, and it provides physicians with immediate information on the level of frailty of patients. In addition, digital technology might the advantage of saving time and processing and store the data instantly. Care should be taken with older patients who are unfamiliar with the use of digital devices, as they may require human assistance to fill the scale, or they may prefer to use the traditional paper scale. Despite these limitations, the eEVA-13 is an effective and innovative health support system for both family caregivers and patients. Certainly, the development of other PROms collected by digital devices is a promising avenue to facilitate geriatric assessment.

## Abbreviations

eEVA-13: Echelle électronique de vulnérabilité des ainés-13
EVA-13: Echelle de vulnérabilité des aînés-13
PROm: patient-related outcomes measure
VES-13: Vulnerable Elders Survey-13

## Data Availability

The data sets generated and analyzed during this study are available from the corresponding author on reasonable request.
